# Cultural and Institutional Factors Driving Severe Repetitive Flood Losses: Insights From the Jersey Shore

**DOI:** 10.1111/risa.70091

**Published:** 2025-08-10

**Authors:** Laura Geronimo, Will B. Payne, Clinton J. Andrews, Elisabeth A. Gilmore, Robert E. Kopp

**Affiliations:** ^1^ NOAA Budget Office Silver Spring Maryland USA; ^2^ E.J. Bloustein School of Planning & Public Policy Rutgers University New Brunswick New Jersey USA; ^3^ Department of Civil and Environmental Engineering Carleton University Ottawa Ontario Canada; ^4^ Department of Earth and Planetary Sciences Rutgers University Piscataway New Jersey USA

**Keywords:** coastal adaptation, fiscal federalism, flood risk, political economy, public administration

## Abstract

Decisions about how to respond to coastal flood hazards often involve disagreements over resource allocations. In the United States, large intergovernmental fiscal transfers have enabled rebuilding in areas that experience severe repetitive losses. This case study focuses on Ortley Beach, a barrier island neighborhood in Toms River, New Jersey, to examine the process of rebuilding after Superstorm Sandy in 2012 and competing visions for the future. A decade later, we conducted 32 key‐informant interviews—including residents and local, state, and federal officials—to examine how values, worldviews, and beliefs shape preferences for coastal risk reduction strategies. A central debate was whether public resources should support staying or leaving the island. Key concerns included the economic impacts of strategies on household and public finances, the effectiveness of strategies to mitigate future flood damages, and fairness in the distribution of costs and responsibilities. Conflicts emerged in how stakeholders framed their preferences. Local officials tended to hold more individualistic–hierarchical worldviews, weaker beliefs in climate science, and favored actions to protect high‐value properties to preserve the tax base while externalizing costs. In contrast, some residents and most state and federal officials held more community–egalitarian worldviews, stronger beliefs in climate science, and preferences for long‐term adaptation strategies to reduce risk, including property buyouts. Responding to the primary concern about economic impacts, we recommend enhancing individual and local financial resilience to climate and political shocks by diversifying municipal revenue streams, encouraging proactive risk‐based planning, exploring innovative insurance models, and better accounting for the long‐term costs of rebuilding.

## 1 Introduction

As climate impacts intensify, so do debates about how to respond. Coastal areas are on the frontlines of sea level rise and intensifying storm surge (Oppenheimer et al. 2019). Billions of people and trillions of dollars in assets have agglomerated along coastlines (Pörtner, Roberts, Adams, et al. 2022), where proximity to water is both amenity and threat (Bin et al. 2008). Policymakers face difficult decisions about prioritizing public resources for coastal adaptation, including which communities to protect, what areas to abandon, and how to distribute costs (Freihardt et al. 2024; Mach and Siders 2021; Penning‐Rowsell and Priest 2015). These decisions are further complicated by the political polarization of climate science and policy (Van Boven et al. 2018).

Coastal adaptation poses significant governance challenges due to diffuse climate impacts and longtime horizons (Pörtner, Roberts, Tignor, et al. 2022). Financing is challenging due to high upfront investments, long‐term benefits, and the need for coordination across levels of government (Bisaro et al. 2020). A common post‐disaster response is to “build back better” (Clinton 2006), but there are diverse interpretations of what “better” means (Benge and Neef 2020). Some interventions may exacerbate vulnerabilities (Sovacool et al. 2015)—a situation known as maladaptation (Macintosh 2013) or response risk (Reisinger et al. 2020). Characterizing trade‐offs is a value‐laden process that requires moving beyond techno‐managerial approaches (Lasswell 1971; Mach and Siders 2021; Sovacool et al. 2015).

Building on prior research (Mallette et al. 2021), we examine how diverse stakeholders prefer to distribute public resources for disaster recovery and adaptation. Our case focuses on Ortley Beach, a barrier island neighborhood in Toms River, New Jersey, that has repeatedly rebuilt despite severe repetitive flood losses. Through in‐depth interviews with 32 key‐informants—including residents, and local, state, and federal officials—we characterize how adaptation preferences relate to beliefs, values, and worldviews. Using values‐informed analysis and the cultural theory of risk (CTR), we analyze competing narratives to understand how stakeholders frame risk and responsibility in repetitive‐loss areas.

Our findings highlight cultural and institutional factors that drive rebuilding in risky areas, including attachment to certain lifestyles and local fiscal dependence on property taxes. This work contributes to research on fiscal federalism and the political economy of climate adaptation, or how the struggle over power and resources may yield inequitable outcomes (Adger, Lorenzoni, et al. 2009; Gotham 2016; Siders 2022; Sovacool et al. 2015; Tanner and Allouche 2011). The rest of this section defines key terms and presents analytical frameworks, their applications, and insights.

### 1.1 Defining Values, Beliefs, and Worldviews

Social scientists have long explored how attitudes, values, and beliefs shape public preferences (Feldman 1988). Research on climate change finds that these factors influence perceptions of risk, responses to hazards, and preferences for adaptation strategies (Dunlap and Brulle 2015; Kunreuther and Slovic 1996; Leiserowitz 2006). However, when comparing studies, terms like “beliefs,” “values,” and “worldviews” are sometimes used interchangeably (Mallette et al. 2021), despite important distinctions. Helgeson et al. (2022) define *beliefs* as people's understanding of how the world works—for example, whether global warming is human‐caused or how fast sea levels are rising. *Values* are “what matters to people.” Values frame beliefs and help simplify complex, uncertain situations. Values may include broad principles like freedom or fairness, or specific concepts like home and family (Helgeson et al. 2022). Worldviews are *sets* of beliefs, values, and assumptions that describe reality (Koltko‐Rivera 2004), or the interpretive lens through which people see the world (Miller and West 1993). Worldviews usually lie along a continuum, for example, ranging from “individualist’ to ‘solidarity” (Kahan 2012).

### 1.2 Values‐Informed Analysis and the CTR

Managing climate risks involves evaluating future outcomes, making values central in decision‐making. This raises the normative question of *whose* values should guide policy (Helgeson et al. 2022). Recognizing tensions among competing value systems, researchers have developed frameworks that center values in climate risk management. We refer to this broader body of work as values‐informed analysis. In New Orleans, for example, Bessette et al. (2017) introduced the methodology of Value‐Informed Mental Modeling (ViMM) to elicit people’ values alongside their risk perceptions and adaptation preferences (Bessette et al. 2017). They found that people prioritized economic growth, protecting ecosystems, and cultural preservation. Similarly, Cooper et al. (2022) found that personal finance, place attachment, and material or emotional loss inform flood adaptation preferences in Pennsylvania.

The “cultural theory of risk” complements values‐informed analysis by linking risk perception to worldviews, suggesting that individuals form beliefs that reinforce their “cultural way of life” (Douglas and Wildavsky 1982). Individualists, for example, may downplay environmental risk tied to industries like oil and gas if those risks may lead to restrictions on their freedoms (Douglas and Wildavsky 1982; Kahan 2012). Conversely, those with egalitarian worldviews are more likely to acknowledge these risks because they see them as unjust social disparities (Douglas and Wildavsky 1982; Kahan 2012). More recently, CTR has been applied to climate adaptation to understand how diverse worldviews shape adaptation preferences (McNeeley and Lazrus 2014).

### 1.3 Enriching Coastal Adaptation Science Through Values‐Informed Analysis and the CTR

This work draws from various strands of literature within coastal adaptation science, including decision analysis (Haasnoot et al. 2013; Hadipour et al. 2020; Lawrence et al. 2019; Martin et al. 2022; Siders and Pierce 2021), sociology and psychology (Bonaiuto et al. 2016; Dietz et al. 2007; Leiserowitz et al., 2021; Meyer and Kunreuther 2017; Stedman 2002), political economy (Elliott 2021; Gotham 2016; Logan and Molotch 1987; Sovacool et al. 2015), fiscal federalism (Catalano et al. 2020; Kousky 2018; Miao et al. 2021; Oates 2005), and multilevel governance (Bisaro et al. 2020; Corfee‐Morlot et al. 2011). Coastal adaptation science has evolved from technical analyses quantifying the costs and benefits of risk reduction (Narayan et al. 2016; Penning‐Rowsell 2005; Turner et al. 2007) to sociopolitical research on the institutional, legal, and social barriers to action (Barnett et al. 2013; Muñoz‐Sevilla et al. 2018; Oberlack 2017; Rahman et al. 2023; Rasmussen et al. 2021; Van Boven et al. 2018).

Our work contributes to this literature by addressing the polarization of climate policy and the need to understand why different groups support or oppose certain strategies (Brink and Wamsler 2019; Hulme 2009; Mallette et al. 2021). Public buy‐in is critical for successful policy implementation, making it essential for planners and policymakers to understand stakeholder values and preferences (Areia et al. 2023; Cash et al. 2003; Cooper et al. 2022).

Value‐informed analysis and CTR enrich coastal adaptation science by providing an analytic framework to understand diverging public preferences. Prior studies identify values underpinning resistance to relocation from risky areas, such as place attachment (Adger, Dessai, et al. 2009; Bonaiuto et al. 2016; Bukvic et al. 2022). In coastal contexts, place attachment stems from aesthetic and recreational benefits, emotional ties, heritage, social networks, and cultural traditions (Mallette et al. 2021). Others oppose retreat due to attachment to property, property values, and entitlement to property rights (Anderson 2022). Understanding these factors enables policymakers to design adaptation plans that appeal to diverse value systems. For example, in the Rhine Delta, Dutch policymakers developed adaptive pathways tailored to hierarchical, egalitarian, and individualistic worldviews (Haasnoot et al. 2013).

Our New Jersey case study adds empirical evidence by comparing how residents and different government actors prefer to prioritize public resources for coastal adaptation a decade after Superstorm Sandy. The analysis highlights the social and political dimensions of decision‐making in areas experiencing severe repetitive losses—challenges that are relevant to other frontline communities. Given limited research comparing residents' preferences with those of decision‐makers, we explicitly examine how residents—the subjects of adaptation decisions—prioritize adaptation differently from the institutions that allocate resources.

## 2 Background

Ortley Beach—a hotspot for coastal erosion—exemplifies broader societal concerns about how to allocate disaster recovery funds in repetitive‐loss regions (Glavovic et al. 2015; de Koning and Filatova 2020; Elliott 2017; Kousky et al. 2023; Siders 2022). While located within the larger mainland municipality of Toms River, New Jersey, this neighborhood sits on a strip of barrier island less than 10 feet above sea level. With a year‐round population of approximately 1500 (2022 ACS 5‐year estimates), the population of Ortley Beach explodes in the summer to accommodate vacationers and secondary homeowners. Most of the residential properties (65% as of 2022) are seasonal or vacation homes.

Superstorm Sandy devastated Ortley Beach in 2012 when storm surge breached the engineered dune system, destroying 200 homes and substantially damaging over one‐third of the housing stock (Maser Consulting et al. 2014). The neighborhood was almost completely rebuilt with millions in state and federal funds for insurance payouts, home elevations, and infrastructure repairs (FEMA 2023; NJOIT 2023). While most aid programs are statutorily obligated to target owner‐occupied homes, 78% of secondary home applicants received FEMA assistance (Halpin 2018). Despite chronic flooding and erosion, development pressures and home prices remain high.

Prior empirical studies on Ortley Beach focus on structural and economic interventions, like storm surge impacts (Xian et al. 2015), structural vulnerability (Hatzikyriakou and Lin 2017), optimal housing elevation to reduce flood losses (Xian et al. 2017), damages to secondary homes (Cheong 2018), and the impacts of updates to National Flood Insurance Program (NFIP) premium rates (Zhang et al. 2022). However, less is known about how the community rebuilt, what people want to see happen in the future, and why. A values‐informed approach illuminates conflicts over public resources in repetitive‐loss coastal areas, and the social and economic costs of rebuilding.

## 3 Methods and Data

### 3.1 Study Participants

We conducted 32 key‐informant interviews, including 15 residents, nine local municipal officials, five state officials, and three federal officials. Residents were mainly recruited through the Ortley Beach Voters and Taxpayers Association (OBVTA) and snowball sampling. Relevant state and federal officials were identified based on roles in coastal risk management and program implementation, as well as snowball sampling.

At the time of the interviews, the local government was Republican, and the state and federal governments were Democratic. Local officials included the mayor, township administrator, council member, township engineer, township planner, code enforcement, and emergency management. The state officials held leadership positions in the New Jersey Department of Environmental Protection (NJDEP), including the Chief Resilience Officer, and senior project or environmental engineering positions within the Division of Coastal Engineering. We also interviewed leadership in the New Jersey Office of Emergency Management (NJOEM). Federal stakeholders occupied leadership positions in FEMA Region II.

Residents were chosen for their deep community knowledge and represented diverse community organizations, including the OBVTA, Friends of Ortley Beach, Toms River Environmental Commission, Toms River Green Team, and the New Jersey Organizing Project. Several residents trained as Coastal Stewards through the Rutgers Environmental Certification Program. One resident is a volunteer for the Zoning Board of Toms River. Residents also represented diverse occupations, including real estate agents, small family business owners, and schoolteachers.

Most residents were primary homeowners, White, over the age of 50, and long‐term residents of Ortley Beach. Incomes ranged from $60,000 to over $200,000, or middle to upper income. While the sample reflects the general sociodemographic profile of Ortley Beach, the limited sample size means that certain groups may be underrepresented, such as renters, secondary homeowners, low‐income groups, non‐White populations, and younger families (see Supporting Information for demographic summary statistics).

Interviews lasted 45–60 min. Most interviews with locals took place in person in June 2022. Interviews with state and federal officials took place via video conference between June and August 2022. Follow‐ups occurred March–June 2023. Interviews were recorded and transcribed with the consent of participants, per IRB protocol. A total of 21 participants completed a voluntary questionnaire.

### 3.2 Interview Protocol

We used a semi‐structured interview protocol adapted from Bessette et al. (2017) and tailored to stakeholder type (see Supporting Information). For residents, interviews began with questions about what brought them to the neighborhood, experiences with flooding, and post‐Sandy recovery. Most of the interview focused on eliciting preferences for federal and state investments in coastal risk reduction strategies. Interviews with decision‐makers were tailored to their specific role: decision‐makers were prompted to talk about their responsibilities, how they make decisions, and preferences for state and federal investments.

Given their familiarity with the local context, residents and local officials were also invited to participate in a voluntary questionnaire, which included more structured questions about preferences toward strategies, worldviews, beliefs about climate change, and sociodemographic information. The questionnaire drew on local and regional hazard mitigation plans and strategic recovery reports to inform the types of strategies included in the interview protocol. First, we presented interviewees with maps of the study region that show impacts from Sandy, including a storm surge overlay, areas that experienced substantial damage, and areas that have experienced repetitive and severe flood losses. After discussing the maps, we presented interviewees with nine coastal risk reduction strategies. The protocol included illustrative diagrams of each strategy. Using a 6‐point Likert scale, respondents indicated how strongly they agreed or disagreed with whether federal and state resources should be invested in each strategy in their community. Participants were encouraged to identify other strategies not included in the protocol. The protocol included a series of follow‐up questions about why interviewees held certain preferences. To measure worldviews, we adapted two attitudinal Likert scales based on the CTR literature (Kahan 2012) and updated per feedback from pilot interviews. The first scale is “Individualism” to “Solidarity” and the second ranges from “Hierarchy” to “Egalitarianism.” We prompted participants to indicate their level of agreement on five worldviews statements, updated per recommendations from pilot interviews. The protocol also included questions to measure participants’ beliefs about scientific consensus on climate change, modeled on prior studies in climate risk perception (Leiserowitz 2006).

### 3.3 Analytic Methods

Analysis involved processing questionnaire data (*n* = 21) and interview transcripts (*n* = 32), focusing on relationships between preferences for strategies, beliefs on climate change, values, and worldviews. Questionnaire data on preferences were recoded to develop a continuum of support for strategies to *stay* or *leave* floodplains. Specifically, preferences were coded on a continuum ranging from −3 (*strongly disagree*) to +3 (*strongly agree*). For the *leave*, the scale reflects participants’ level of agreement with property buyouts as a strategy, given that this is the principal method for managed retreat (Miao and Davlasheridze 2022). For *stay*, the scale reflects the average score the participant responded for interventions to protect and accommodate flooding (e.g., beach nourishment, seawalls, home and roadway elevations). We also developed a continuum summarizing belief in scientific consensus on climate change, ranging from strong to weak. For worldviews, we developed a continuum from an individualistic/hierarchical worldview to a community/egalitarian worldview. We then analyzed the relationships between preferences for strategies, beliefs, and worldviews.

For analysis of the transcripts (*n* = 32), we applied both deductive and inductive coding methods. Modeling after elements applied in ViMM studies, we coded different concepts, themes, or “nodes” and their co‐occurrences. Using NVivo software, we coded for three distinct categories: strategies, values, and risk factors. These methods enabled systematic exploration of how values and beliefs about risk relate to preferences for strategies. We also allowed new codes and categories to emerge inductively. For consistency purposes, one researcher conducted iterations of coding to identify, categorize, and consolidate codes. The result is a hierarchical coding system of categories and concepts linked to specific strategies, the central unit of analysis. While more involved ViMM studies include coproducing complex mental models with diverse stakeholders, we focused this study on factors that shape preferences for public investments.

While the questionnaire and coding are useful tools for synthesis, they do not always capture nuances embedded in the text, such as the direction of sentiment, the context framing rationales, or how groups and individuals construct their storylines. Therefore, we use discourse analysis to synthesize, characterize, and compare the competing narratives, identities, and power relations embedded in the transcripts (Koteyko and Atanasova 2016; Schlosberg and Collins 2014). We examine how preferences vary across stakeholder types, how stakeholders frame preferences, and how preferences are grounded in conflicting beliefs, values, and worldviews. We select illustrative quotes to illustrate points of tension or conflict.

## 4 Results

Stakeholders exhibited diverse values, beliefs, and worldviews when discussing coastal flood risk and preferences for public spending on risk reduction. Long‐term residents were torn about whether federal and state subsidies should support efforts to stay or leave, citing concerns about how subsidies may create perverse incentives, distort coastal housing markets, and catalyze gentrification. Municipal officials favored rebuilding and protecting exposed barrier island communities despite obvious risk, referencing the need to preserve tax revenues from high‐value properties. State and federal officials expressed concern about shouldering an increasing cost burden of rebuilding in repetitive‐loss areas. These conflicting narratives shape which strategies get implemented and who benefits from public investments.

### 4.1 Questionnaire Results (*n* = 21)

#### 4.1.1 Preferences for Strategies

**FIGURE 1:**
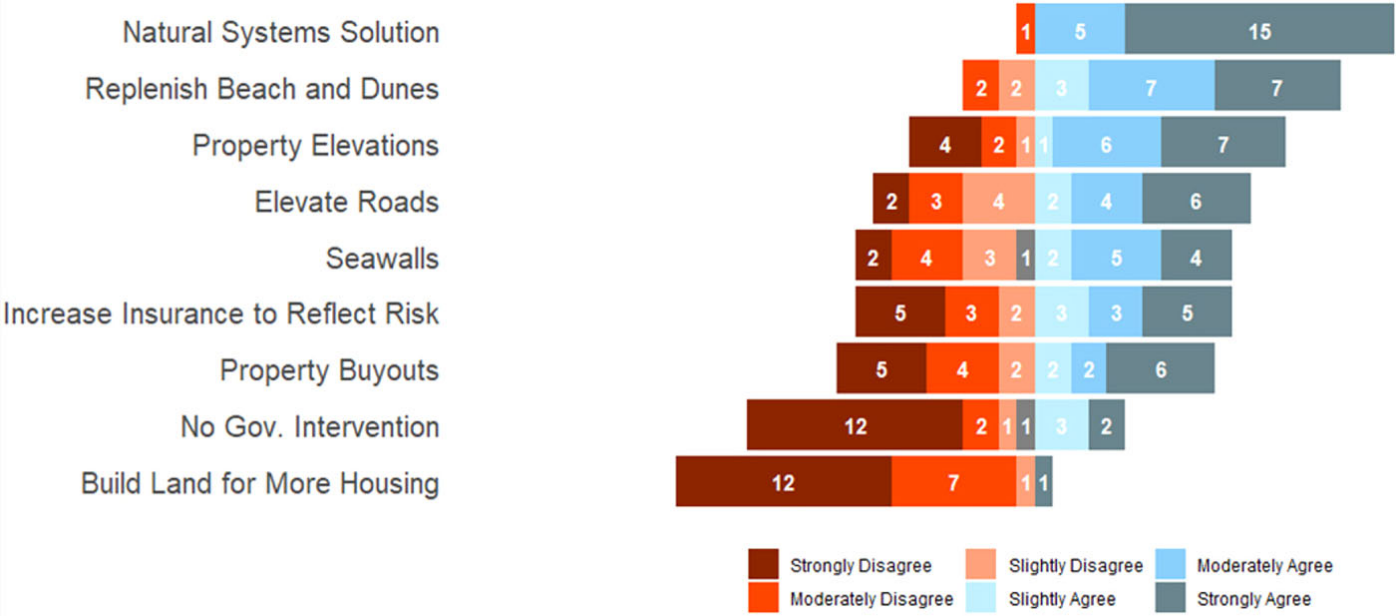
Responses to question, “Please indicate how strongly you agree or disagree with whether federal and state resources should be invested in each strategy within the context of Ortley Beach, NJ.”

Most respondents expressed strong or moderate agreement with restoring natural systems and replenishing beach and dunes (Figure 1). There was strong disagreement with building land for more housing and no government intervention. Among the other strategies evaluated, there was a mixture of agreement and disagreement. Respondents were about evenly split regarding support for buyouts. Most respondents (14 out of 21) supported property elevations.

In addition to the strategies included in the questionnaire, new strategies emerged from the interviews. These included other hard infrastructure interventions like bulkheads, jetties, and storm surge barriers. These strategies were generally rated favorably, though there were mixed views on who should pay for them: people tended to think that bulkheads should be a private investment, while state and federal agencies should finance jetties and larger structures.

Residents also emphasized other “soft” strategies to mitigate risk and achieve fair outcomes when rebuilding, including the important role of social networks and community‐based organizing. Many residents identified these networks as crucial first responders and long‐term support systems.

Respondents discussed another set of strategies that we broadly categorized as municipal powers. These include building codes, permits, zoning, planning, and enforcement. Municipal officials emphasized that municipal powers are crucial to ensuring the effectiveness of existing strategies like property elevations: without code enforcement, the effectiveness of home elevations could be compromised.

#### 4.1.2 Beliefs and Strategies

**FIGURE 2:**
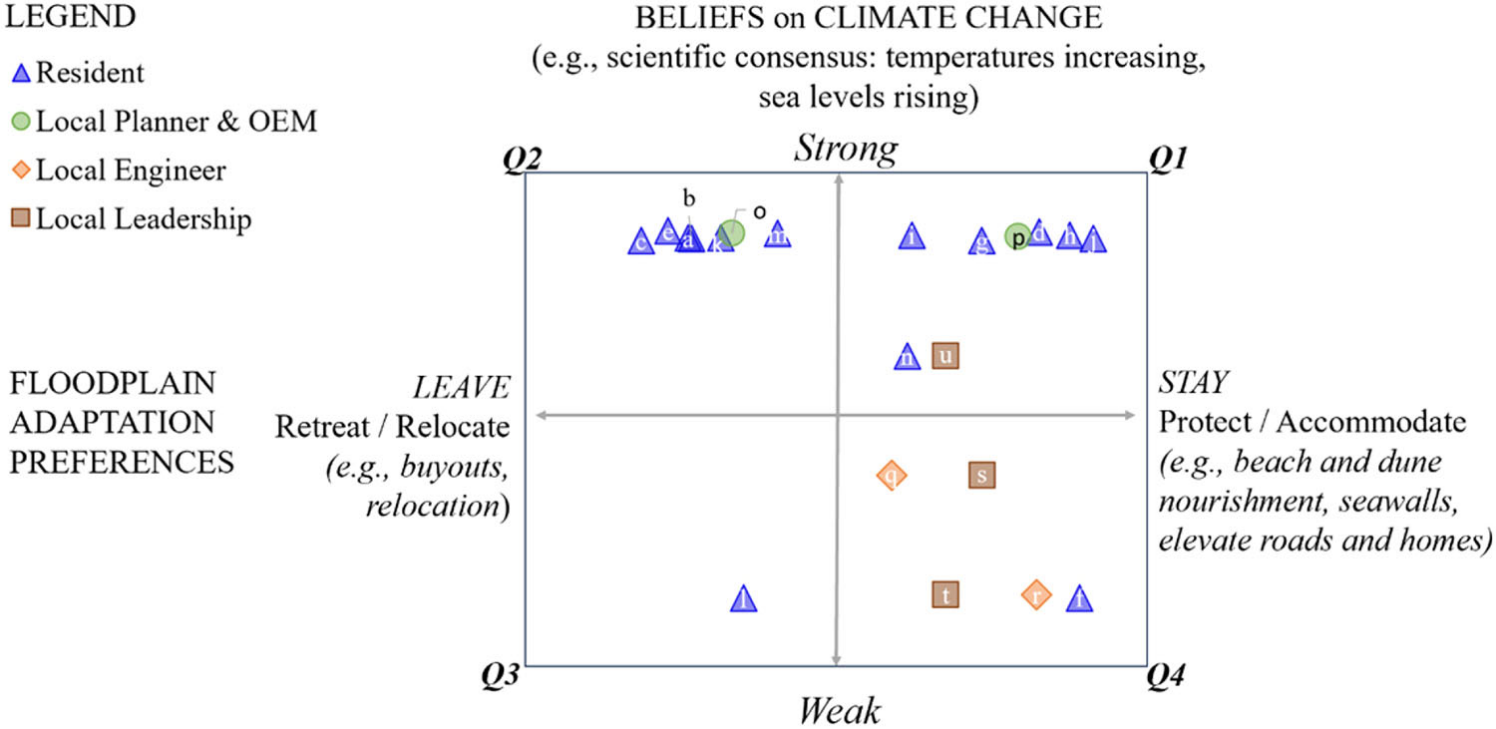
Mapping preferences for strategies to “stay” or “leave” against beliefs on climate change, by stakeholder type. Each dot represents an individual with a reference letter. The colors/shapes represent the type (e.g., blue triangles are residents, while green circles are local planners or Office of Emergency Management [OEM]). The letters enable comparison with Figure 3. For example, resident “b” has a preference to invest in strategies to leave the island and also demonstrates a strong belief in the scientific consensus on climate change and more community–egalitarian worldviews.

Stakeholders held a range of beliefs about climate change (Figure 2, vertical axis), which we mapped against preferences to *stay* or *leave* exposed floodplains (horizontal axis). Most residents strongly believed in the scientific consensus on climate change, though they varied in their preferences on how to respond. Conversely, local officials in leadership positions (mayor, council member, administrator, engineers) held weaker beliefs in the scientific consensus on climate change. Four out of the five participants in these positions incorrectly believe that scientists are divided on the statement that “sea levels are rising at an increasing rate.” They demonstrated skepticism toward climate models and FEMA flood maps, citing contradictions with local tide gauge records and observations. These officials favored actions to *stay* in floodplains through protection and accommodation strategies.

Federal and state officials, by contrast, held strong beliefs in the scientific consensus on climate change and preferred strategies for managed relocation. As one state official stated, “I have a strong opinion that elevating and putting people back in the floodplain isn't appropriate. Why not spend the resources to really remove the risk?”

#### 4.1.3 Worldviews and Strategies

**FIGURE 3:**
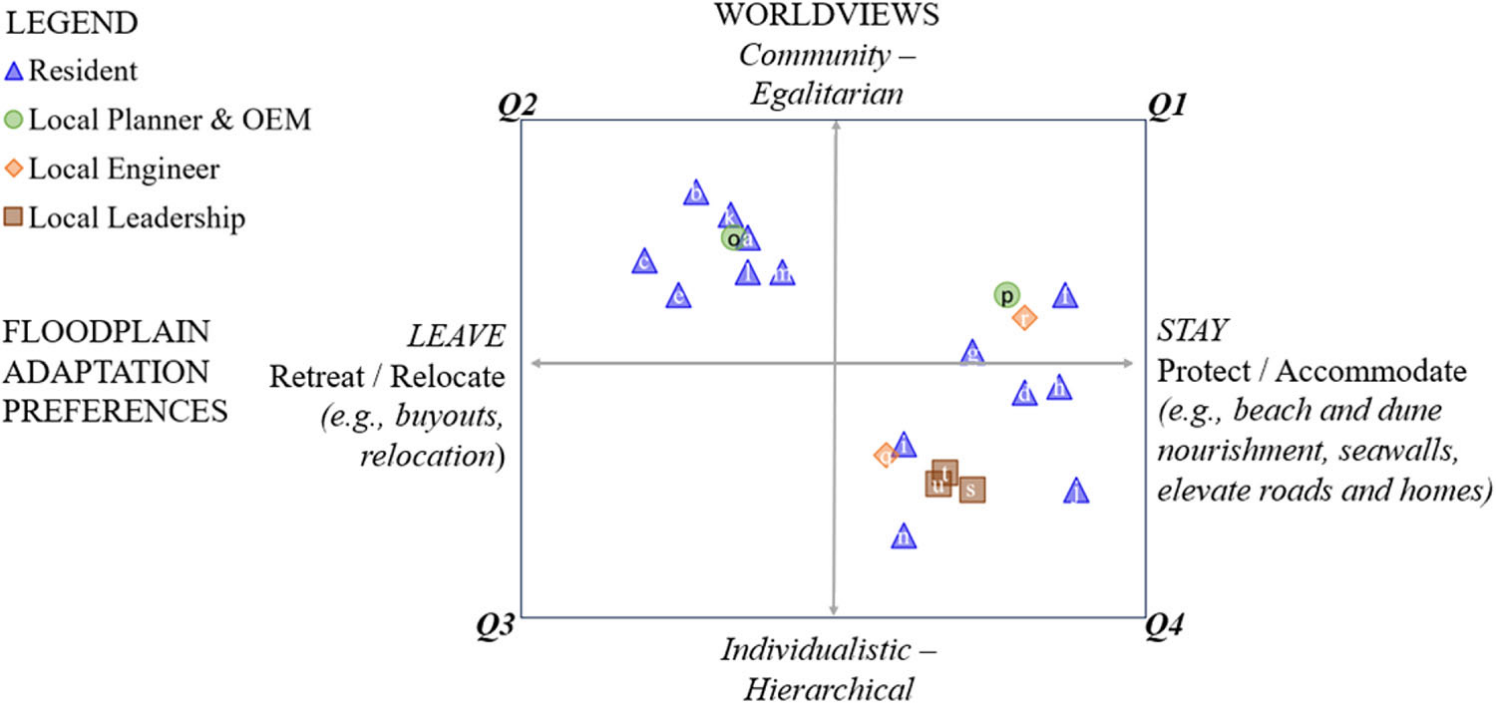
Mapping preferences for strategies to “stay” or “leave” against worldviews, by stakeholder.

When mapping worldviews (vertical axis, Figure 3) against preferences, a pattern emerged: respondents with more community/egalitarian worldviews showed stronger support for strategies to leave the floodplain like buyouts. In contrast, those with more individualist/hierarchical worldviews tended to favor investments in protection and accommodation strategies. This pattern is demonstrated by clustering in Q2 and Q4. Notably, most local leadership and engineering officials cluster in Q4.

Interview data suggest that federal and state officials would likely cluster in Q2. Federal officials evinced concern for the fair distribution of resources at a national level, indicating a more community/egalitarian worldview. For example, one official expressed concern that the point ranking system for risk reduction projects was tilting resources toward the Eastern seaboard, away from other deserving areas.

### 4.2 Transcript Analysis (*n* = 32)

To complement the questionnaire findings, we conducted in‐depth transcript analysis. First, we draw methods from values‐informed analysis to assess the prevalence and frequency of values in relation to strategies. Then we used discourse analysis to characterize the context and rationales surrounding stakeholder preferences for strategies, illuminating key areas of conflict.

#### 4.2.1 Values‐Informed Analysis: Prevalence and Frequency of Values Relating to Strategies

Like prior studies (Bessette et al. 2017), we coded transcripts for values discussed in relation to strategies. Note that 10 clusters of values emerged. We identified and defined these values and described their prevalence, or the percentage of participants who evince this value (see Supporting Information). The most prevalent value that all participants discussed was economic impacts (100%), reflecting its importance. The next most prevalent values were procedural equity (97%), followed by distributional equity (80%), effectiveness–protection (73%), and responsibility or cost share (62%). We coded “change in community character” and “place attachment” separately because the former reflects observations on changes in the community, while the latter is about what the person values about place. Respondents discussed these values with similar prevalence (57% and 54%, respectively). The least prevalent values were intergenerational equity (15%) and environmentalist values (10%).

#### 4.2.2 Discourse Analysis: Characterizing Competing Narratives on the Proper Course of Action

Building on the coding and quantitative analysis, the following section summarizes findings from an in‐depth study of the transcripts using discourse analysis. We characterize the complex relationships between values, beliefs, and worldviews and preferences for coastal adaptation strategies. We summarize the competing narratives articulated by residents, local officials, and state and federal officials, drawing on their lived experiences recovering from Superstorm Sandy.

##### 4.2.2.1 Resident Perspectives

Key‐informant interviews with 15 residents of Ortley Beach reveal that residents are torn about the best use of public resources for recovery and adaptation. Residents are most concerned about economic impacts, neighborhood effects, procedural and distributional equity in accessing government resources, and the effectiveness of risk reduction strategies.

Regarding economic impacts, residents are concerned about personal finances and efficient use of public funds for long‐range adaptation. Residents stressed the steep costs of post‐Sandy rebuilding and home elevation, with some incurring over $100,000 in out‐of‐pocket expenses. Residents also mentioned drastic increases in insurance costs after Sandy. Residents mentioned how a first “wave” of neighbors was displaced by the economic shocks: “maybe more than half the people left this island.” Several residents mentioned that if another Sandy occurred, they would be caught in a second wave of displacement: “if this happens again. I'm gone.”

Residents also described drastic changes in community character since Sandy, with small one‐story bungalows being converted into multimillion‐dollar four‐story homes. Many homes were purchased by investors and converted to vacation rentals or secondary homes. People who could afford to stay often used rebuilding as a chance to build out larger homes. “The whole vibe of the place is different,” said one long‐term resident. “I recently had a home sell for $3 million, which has never happened,” mentioned a realtor. Others expressed concerns about “increasing property taxes” or “climate gentrification.” Residents mentioned “overdevelopment” as a serious threat to the community, and expressed concern about bottlenecks on the main bridge during emergencies.

Regarding procedural and distributional equity, many residents described barriers to navigating the complex disaster aid bureaucracy. For example, several residents felt that there was a degree of arbitrariness in how damages were assessed for flood insurance claims. One resident hired a public adjuster to assess the damages, saying it was “the best thing I did, because I ended up getting every drop of money from FEMA,” while neighbors got less. Overall, residents described “emotional trauma” associated with Sandy and the process of recovery and rebuilding.

Residents described concern about the effectiveness of interventions and implications for public safety, evincing concern about how certain strategies would stand up to future climate impacts. Regarding building elevation requirements, residents felt that this could reduce flood risk but may increase risk associated with wind: “Every single time now that it's windy out, I can feel the house moving.” Regarding beach nourishment, several residents believed it is essential to protect homes, but most expressed frustration with constant erosion and the rising costs of maintenance. One resident said “there is a limit to how much this dune replenishment is going to work. The amount of sand we have lost in the past five years is staggering.” Another resident commented that “it's the definition of insanity: doing something over and over again and expecting a different result.”

Given concerns about the effectiveness of beach nourishment and home elevations, several community leaders have advocated for property buyouts. However, residents mentioned that municipal representatives discouraged buyouts after Sandy for fear of losing their tax base. One resident said that there is a political disincentive to implement buyouts: “The reason that Toms River wouldn't listen to us was because they wanted those ratables: If they get that $30‐$40 M worth of ratables, then the taxes stay low, and they get reelected. It's a huge conflict of interest.” While some residents disagree, others view buyouts as an equitable, economical, and effective strategy: “I strongly agree. Some people have lived here 40–50 years. You have to give them a fair way out. What's more expensive: spending billions of dollars every time there's a storm? Or a billion dollars to get everybody off the island and be done with it?”

Overall, residents mentioned the lack of foresight at the municipal level as a risk to the community, reflecting conflicts in beliefs over the severity of future impacts. Some residents emphasized the need for long‐range planning: “You still want your grandkids or your future people to enjoy the beach, enjoy the natural environment. But if we don't do something to plan it out, it's just going to be a bunch of homes underwater.”

##### 4.2.2.2 Local Official Perspectives

In‐depth interviews with nine local officials revealed a different set of priorities, grounded in conflicting beliefs, values, and worldviews. The mayor and lead engineers were skeptical of science on sea level rise, optimistic about engineering solutions, and adamant about rebuilding and protecting high‐value homes to support the tax base. In contrast to several residents who are ready to entertain relocation as a long‐range strategy, most municipal officials interviewed were firmly opposed to buyouts. Top municipal officials viewed this strategy as economically prohibitive on the barrier island because property values are so high: “Blue Acres [a state buyout program] had $15 million total. That's like one street in Ortley Beach.” Municipal officials also did not view buyouts as politically or legally feasible: “It's America. People have property rights.” They also disfavored turning high‐value properties into empty lots that would then require maintenance.

Instead of buyouts, top municipal officials strongly supported directing federal and state investments to keep homes in place. Immediately after Sandy, municipal officials relaxed building code standards to help people to quickly return to their homes. Officials granted variances to allow people to improve and enlarge their homes as they rebuilt and elevated, adding to the development pressure. Officials expressed confidence in property elevations if building codes are enforced. The former town mayor lobbied for federal assistance to help secondary homeowners repair, elevate, and protect their homes: “Everybody's paying the same taxes, so they should get the same benefits.”

Toms River municipal officials also expressed strong support for investing public resources in complementary actions at the community level, such as roadway elevations and beach nourishment, especially if costs are assumed by the state and federal government. Municipal officials have successfully negotiated to reduce or eliminate the local cost share for dune replenishment in recent replenishment cycles. In one round of beach replenishment for neighboring Seaside Heights, Ocean County absorbed the full 25% local cost share for the $6 million project. The former mayor of Toms River argued that since the public uses the beaches and they are essential to the coastal economy, the cost share for beach replenishment should be borne at the federal and state level. Referring more broadly to the billions of federal dollars appropriated by the Infrastructure Investment and Jobs Act, one official from Seaside Heights said, “Now it's a race to the money. And Seaside is in the race.” Municipal officials also favored more ambitious state and federally funded measures like storm surge gates at key inlets, such as those proposed in the $16 billion NJ Back Bay Coastal Storm Risk Management Plan (USACE 2021).

In the case of Toms River, people occupying positions of power—such as the mayor and lead engineering positions—reflected the values and interests of secondary homeowners and investors. These values often superseded the values and interests of long‐term primary homeowners and renters. The former municipal engineer embraced the changes: “One good thing about Sandy is it kind of flushed out all the problem areas.” However, not all local officials shared the same views. The township planner felt that rebuilding Ortley Beach may have been an error caused by a combination of federal and state funding and market forces: “one of the mistakes that was made was putting all the infrastructure back where it was.” Before the town planner could have a say, the “federal government jumped into the breach.”

##### 4.2.2.3 Federal and State Official Perspectives

In contrast to municipal leadership, federal and state officials expressed greater concern about subsidizing redevelopment in severe‐repetitive‐loss areas and a preference for considering alternative long‐range solutions like relocation. However, state and federal officials described barriers to implementing this vision. These barriers include inflated expectations for state and federal support, institutional rules for program implementation, market responses to federal and state interventions, and conflicts over perceived effectiveness of protective infrastructure.

One key challenge is the expectation among local officials for substantial state and federal funding to rebuild and sustain flood‐prone communities, raising concerns about a culture of entitlement and dependency on such transfers. Local governments sometimes expect “extraordinary aid,” but limited resources make it impossible to protect all communities. State and federal officials expressed concern about who takes responsibility for maintaining infrastructure over the long term: “at some point, we've all got to have a little bit more skin in the game: everybody from the property owner to the local government.”

Federal officials described how program rules and regulations determine what level of government has discretion over resource distribution. FEMA Region 2 officials described differences between older programs, like the Hazard Mitigation Grant Program (HMGP) authorized in 1988 by the Stafford Act, and newer programs like the Building Resilience Infrastructure and Communities program (BRIC), authorized in 2018 by the Disaster Recovery Reform Act. Under the Trump Administration, the BRIC program has been terminated, and there is reduced spending through the HMGP. Previously, state and local governments had more discretion in programs like the HMGP because “as long as they meet the eligibility criteria, then they're going to get funded.” In contrast, for BRIC, the federal government outlined funding guidelines in the notice of funding opportunity that include incentivizes for projects to allign with federal priorities “like building codes or green infrastructure or equitable outcomes.” In this way, programs like BRIC represented efforts to shift the locus of discretion to higher levels of government, while legacy programs were more locally controlled. The termination and reduction of these programs reflect an interest in devolving hazard mitigation responsibilities to the local level.

At the time of the interviews, billions of dollars of federal funding were flowing to hazard mitigation projects through the Bipartisan Infrastructure Law and the Inflation Reduction Act. Democratic state and federal officials expressed concerns about how interventions might create market distortions. These officials observed that wherever the state and federal entities fund resilience interventions like beach nourishment, the market rapidly catches on: investors capitalize on the improvements, property values shoot up, and housing affordability becomes an issue. “The missing part is immediate and utter gentrification after a massive investment of outside capital. […] After you put an investment of a couple hundred million into a flood abatement system, it spurred an immediate gentrification of downtown….” Officials worry that resiliency interventions might end up benefiting a different population from the one originally targeted.

Finally, state and federal officials expressed concern that climate science is not being sufficiently incorporated into the design and cost estimates for protective infrastructure projects. For example, when discussing the storm surge gates in the NJ Back Bay Plan, state officials at the NJDEP Coastal Division Unit staff said that it is unclear if this technology has been tested in a barrier island environment: “as far as I'm aware, this would be the first direct ocean front storm barrier in the United States.” Officials also questioned what the secondary effects of sea gates might be, including dune breaches or impacts to the tidal flow and health of bays. State officials mentioned that more efforts are needed to integrate sea‐level‐rise projections into the engineering design. Finally, state officials mentioned that funding for the project is not guaranteed and depends on approval from Congress, which may result in delays. Since the change of administration in 2025, state and local governments face a new landscape wherein the federal government is largely rescinding funding and responsibility for local hazard mitigation efforts, generating a whiplash effect where communities dependent on intergovernmental transfers as suddenly stranded on a fiscal cliff.

## 5 Discussion

### 5.1 Institutional and Cultural Barriers to Proactive Climate Adaptation

As climate impacts escalate, so do conflicts about how to distribute public resources for response, recovery, and adaptation (Bisaro and Hinkel 2016; Kousky et al. 2023). Public officials often face difficulties balancing public and private interests (O'Donnell and Gates 2013), particularly in US coastal regions where federal, state, and local governments face the challenge of managing scarce resources, escalating flood risk, and intense development pressures (Wing et al. 2022). Coastal adaptation efforts often become deadlocked by social and political conflict (Rasmussen et al. 2021). Studies that clarify sources of conflict can help identify barriers to proactive action (McNeeley and Lazrus 2014).

The Ortley Beach case study highlights conflicts between residents, and local, state, and federal officials over coastal climate adaptation investments, underscoring tensions along the vertical axis of adaptation governance and finance (Bisaro et al. 2020). Diverging views on climate science, values, and worldviews drive competing narratives on public resource allocation. Overall, the central conflict is whether public resources should support actions to stay or leave high‐risk areas—and who should decide. Local leaders favored rebuilding high‐value properties, reflecting a broader cultural trend of justifying protection measures on the basis of property value rather than human well‐being (Anderson 2022; Cutter et al. 2014). Their values reflected a broader culture of prioritizing the exchange value of homes over their use value, bolstered by urban growth machine politics fueled by boom and bust economic cycles (Logan and Molotch 1987). In contrast, long‐term residents expressed a strong attachment to place but also worried about rising costs of living, neighborhood changes, and safety. Federal and state officials sought to manage fiscal risk and ensure equitable resource distribution. Residents, state, and federal officials supported considering buyouts in long‐range planning. However, these stakeholders have less discretion implementing adaptation actions given institutional barriers and the norm of local control in land use decisions (Kaswan 2014).

Evidence from the study supports the CTR, in which stakeholders adopt values, beliefs, and worldviews that reinforce their desired way of life (Douglas and Wildavsky 1982; Dunlap and Brulle 2015; Elliott 2021; Kahan 2012). Extending this line, we propose a cultural theory of adaptation, where people adopt preferences toward adaptation investments that reinforce their desired lifestyles and externalize costs. This work is especially relevant given capital interests in coastal real estate (Chandra‐Putra and Andrews 2020; Gould and Lewis 2021; Keenan et al. 2018), increased polarization of climate change science and policy (Dunlap and McCright 2011; Jylhä and Hellmer 2020; McCright 2016), and the fiscal incentives and political ideology that influence adaptation decisions (Bisaro et al. 2020; Miao 2019; Miao et al. 2024; Oates 2005). Findings contribute to work on fiscal federalism and the political economy of climate adaptation, or how the struggle over scientific narrative, power, and resources may yield inequitable outcomes (de Koning and Filatova 2020; Sovacool et al. 2015).

### 5.2 A Way Forward: Build Individual and Local Financial Resilience

Economic impacts emerged as the most pressing issue among all stakeholders when considering coastal adaptation strategies. Concerns included the impact on household finances, strain on municipal budgets, and how to prioritize state and federal investments. Thus, one important way to improve climate resilience is by strengthening the financial resilience of individuals, communities, and the public sector (OECD 2022). Below, we discuss options like diversifying municipal revenue streams, supporting proactive risk‐based planning, piloting innovative insurance models, and more fully accounting for the costs of rebuilding. These actions are interrelated and may require innovative financial arrangements and incentives that promote cultural and behavioral shifts (Meyer and Kunreuther 2017).

States and municipalities that rely heavily on property taxes face uncertain fiscal vulnerabilities as climate impacts erode property values and increase the service costs (Gilmore et al. 2022). After New Hampshire, New Jersey ranks second nationally in its reliance on property taxes, which account for 27% of the state's combined state and local general revenues (Tax Policy Center 2024). New Jersey's strong home rule system reinforces this dependence, making it difficult to break away from property‐tax‐based financing (Bruck and Pinto 2007). When disasters damage property, these municipalities become dependent on intergovernmental transfers to balance budgets (Jerch et al. 2023).

In growth‐driven places like Toms River, where local officials have incentives to promote development in high‐risk areas, state policymakers could disincentivize such behavior by tying future intergovernmental transfers—like state and federal aid—to local adoption of risk‐based zoning (Wildasin 2008). Shifting school funding from property taxes to state income taxes could also help decouple school quality from local property values, reducing the incentive to encourage high‐risk development for fiscal reasons (Brunner and Sonstelie 2003). By diversifying municipal revenue streams, policymakers could remove a major political barrier to proactive adaptation actions like buyouts (BenDor et al. 2020; Curran‐Groome et al. 2022).

Relatedly, proactive land use planning can help communities adapt by anticipating impacts and identifying socially acceptable thresholds for adaptive actions like relocation, thereby reducing long‐term economic losses (Adger, Lorenzoni, et al. 2009; Berke and Stevens 2016; Burby et al. 1999; Godschalk et al. 2009; Haasnoot et al. 2013). Planning tools like coastal setbacks, inundation overlay zones, and building design or code enforcement metrics can incrementally reduce exposure to climate hazards (Butler et al. 2016; Hudson and Botzen 2019; Rasmussen et al. 2020).

Insurance also plays a critical role in building financial resilience—if well designed (Kousky 2020). Ideally, flood insurance provides financial protection, prevents post‐disaster economic hardship, and accelerates recovery (Kousky 2019). However, the US NFIP has faced criticism for underpricing risk, incentivizing moral hazard, and providing insufficient coverage (Elliott 2021; Kousky 2018). Emerging insurance models and financial instruments—including parametric insurance, microinsurance, and insurance‐linked securities like catastrophic bonds—offer promise. These tools, especially when linked to proactive land use planning, can provide faster, more inclusive financial protection (Kousky 2022).

This study also highlights the need to revisit standard frameworks for assessing climate adaptation trade‐offs (Siders and Pierce 2021). Benefit‐cost analyses often omit rebuilding costs like household out‐of‐pocket expenses, future federal liabilities, and long‐term protection expenditures (FEMA 2021). Our findings underscore the importance of capturing the full costs of rebuilding in high‐risk areas and how these are distributed across stakeholders (Elliott 2017; Freihardt et al. 2024; Sovacool et al. 2015). Since adaptation cost‐benefit assessments inherently involve value judgments (Siders 2019), decision support systems incorporating sociological methods could help shift from a technocratic approach to a more deliberative, participatory model (Glavovic 2016; Glavovic et al. 2015; Siders 2022). For example, serious games—wherein participants role‐play and negotiate trade‐offs—could help build trust, democratize planning, and socialize adaptation priorities (Flood et al. 2018). Insights from this study provide a foundation for designing such tools, anticipating stakeholder behaviors, and supporting more informed and inclusive adaptation planning. In the absence of consistent federal leadership on climate action, states and municipalities may need to build coalitions and experiment with alternative financial arrangements to improve resilience (Basseches et al. 2022; Shi and Moser 2021). Given the scale of risk, some market‐based risk absorption may be necessary while simultaneously identifying pathways to minimize fiscal shocks (Gourevitch et al. 2023).

## 6 Conclusion

In US coastal contexts like Ortley Beach, New Jersey, there is now a strong culture and political economy of rebuilding despite severe repetitive losses. Current cultural and institutional arrangements reinforce this pattern, creating barriers to transformative adaptation strategies like community‐led relocation (Cooley et al. 2022). The current disaster recovery and adaptation system is characterized by uncoordinated intergovernmental transfers that tilt resources toward rebuilding, a municipal finance system dependent on property taxes that favor investor interests, and the entrenchment of those interests within local government.

Breaking the build–damage–rebuild cycle requires rethinking the fiscal, governance, and social systems that regulate activity in the coastal zone. In some contexts, reducing federal and state subsidies for rebuilding could shift some of the cost burden onto local governments and housing markets. Simultaneously, there is a need to recognize differential vulnerability within coastal communities: individuals have different exposure levels and risk tolerances, and therefore different future needs (Thomas et al. 2019).

When confronted with severe repetitive flood losses and intense development pressure, individuals, neighborhoods, and governing institutions need frameworks for prioritizing scarce resources. Currently, intergovernmental transfers prioritize physical infrastructure projects as a path to resilience. However, the full social and economic costs of rebuilding are still poorly understood. Current fiscal arrangements and reliance on property taxes for local services create perverse incentives for local officials to rebuild in risky areas. Rather than defaulting to the build–damage–rebuild cycle, policies designed to strengthen individual and local fiscal resilience may support more flexible adaptation to climate and political shocks. By combining innovations in municipal finance, proactive land use planning, insurance models, and trade‐off analysis, coastal communities can enhance their financial resilience to these shocks. Evidence from the Ortley Beach case study indicates that it may be possible to build support for these types of proactive climate policies by showing how actions would reduce strain on household and public finances.

## Supporting information




**Supplementary Materials**: risa70091‐sup‐0001‐SuppMat.docx

## Data Availability

Interview transcripts and questionnaire data have been de‐identified per IRB protocol and placed in a repository online in accordance with funder data retention policies. Data are available at https://www.openicpsr.org/openicpsr/project/233521/version/V1/view.
