# Supplementary material for: Cultural and Institutional Factors Driving Severe Repetitive Flood Losses: Insights From the Jersey Shore
Source: Risk Anal. 2025 Aug 10;45(11):3519–32. doi: 10.1111/risa.70091 (PMC12663900; doi:10.1111/risa.70091)

**Cultural and Institutional Factors Driving Severe Repetitive Flood Losses: Insights from the Jersey Shore.**

**Supplementary Materials**

______________________________________________________________________________

# Interview Protocol: Residents

**INTERVIEWS WITH RESIDENTS GUIDE**

**[date, time]**

**Moderator: [name]**

**[location]**

I. WELCOME AND INTRODUCTION

Welcome! Thank you for participating in this interview, which is part of a project to develop a better understanding of preferences for different flood risk management strategies for properties in coastal New Jersey. The purpose of the study is to better understand stakeholder preferences for different types of coastal flood risk management strategies in specific contexts, and their perceptions of the tradeoffs of these different strategies. The study focuses on specific neighborhoods in Toms River, New Jersey, where there has been repetitive damage from flooding and significant investment of federal funds in flood hazard mitigation.

This is one of several interviews carried out by researchers from XXX University with support from the New Jersey Sea Grant Consortium Before we begin, a few points:

- My name is XXX, and I am a PhD Candidate at the XXX at XXX University. My dissertation focuses on understanding how federal funds, like FEMA funds, are implemented to mitigate flood risk in the coastal zone, and the tradeoffs of different strategies applied at the household level. I am very curious to understand your thoughts on this topic.
- This is a semi-structured interview. There is a lot of material to cover in an hour, so I may interrupt you occasionally to keep the ball rolling.
- Have you had a chance to review the consent form? Do you have any questions?
- Participation is voluntary; you can choose to answer, or not; and stop at any time. There are no right or wrong answers.
- We are recording the meeting unless you disagree.
- As indicated in the informed consent, we are conducting confidential or non-confidential interviews. If you are participating in a non-confidential interview, you are willing to allow us to use your names and the names of your organization in the research report. The identifiable information will be stored with your responses. If you are participating in a confidential interview, you are not willing to allow us to use your name or the name of your organization. The identifiable information will not be stored with your responses. Do you consent to a non-confidential interview? Or do you prefer confidential?
- Finally, the interview also involves a survey component, some parts of which we hope to discuss with you. I will not be able to see your screen when you are answering the survey questions unless you decide to share your screen.
- Do you have any questions before we get started? Time limit: 1 hour

**Preamble and Instructions *(1-2 min)***

Again, the point of the interview is to gauge how people, like you, think about the tradeoffs of different flood risk mitigation strategies in your neighborhood and region. We are interested in understanding what you think are the pros and cons of different strategies. When you’re thinking about your answers, try to think about them both from your own personal perspective and also from the perspective of your community. The interview also involves a survey component, some parts of which we hope to discuss with you. I will not be able to see your screen when you are answering the survey questions unless you decide to share your screen.

Finally, I’d also like to get your permission to audio record this interview. Having a recording will help to give me a more accurate understanding of your views. We will transcribe the recording. (*If they chose a confidential interview*) All identifying remarks or information you give us will be cut out, so no one can use your transcript to identify you. Is that ok? *(wait for affirmative).* Do you have any questions before we get started?

1. **General questions: *(10 min)***

Let’s get started with some general background questions.

- 1. Where you are originally from, and how long you have been living in your neighborhood?
  2. What are some things that brought you to this neighborhood?

1. **Experience with flooding: *(10 min)***

I want to learn about your experiences with flooding. Some neighborhoods are experiencing flood problems.

- 1. Do you have concerns about flood risk to your personal property?
  2. ***(****If not fully inferred from 2.1)* Have you experienced flooding to your personal property or in your neighborhood? Under what circumstances?
  - *Prompts:* What types of flooding have you seen: (storm surge, riverine, high tide flooding, heavy rains / stormwater).
  1. Do you have experience with federal disaster recovery programs? (prompt: FEMA insurance and mitigation programs, HUD programs).
  - *Prompts if yes:* What was your experience like navigating access to aid through those programs? Were there any hangups? Where the hangups at the local, state, or federal levels?

1. **Preferences regarding Coastal Risk Management (CRM) Strategies *(20 min)***

Now we’re going to move on to strategies to deal with flood risk in Toms River. The government is spending a lot of money to manage flood risk in coastal New Jersey. I am interested in your thoughts about how government spending should be used to mitigate flood risk at the property and neighborhood level.

- 1. What would you like to see done to deal with flood risk to your property or neighborhood?
  2. Is there anything about (that strategy) that maybe concerns you like or that you don’t like? (Why?) (follow up: What about things that you like about that strategy?)

Now I’d like to get a sense for how you might compare different strategies in the context of your property or neighborhood. I am going to send you a link to a survey that I want you to work through and discuss with me. I am sending the link in the chat now.

If you could just open that link, the first page is the required consent. When you are ready, let me know when you have moved on to the next page with the maps.

Below is a map of Toms River extracted from the 2016 Toms River Local Hazard Mitigation Plan. The map shows FEMA's preliminary flood hazard areas. Data from the National Flood Insurance Program (NFIP) is overlaid on top to show which neighborhoods have experienced repetitive loss or damages from flooding. Areas in yellow represent neighborhoods where there has been repetitive loss, and areas in red are those neighborhoods where there has been severe repetitive loss.

Certain neighborhoods of interest are marked with a black circle, including the barrier island communities of Ortley Beach, Dover Beaches North, and the back bay community of Silverton.


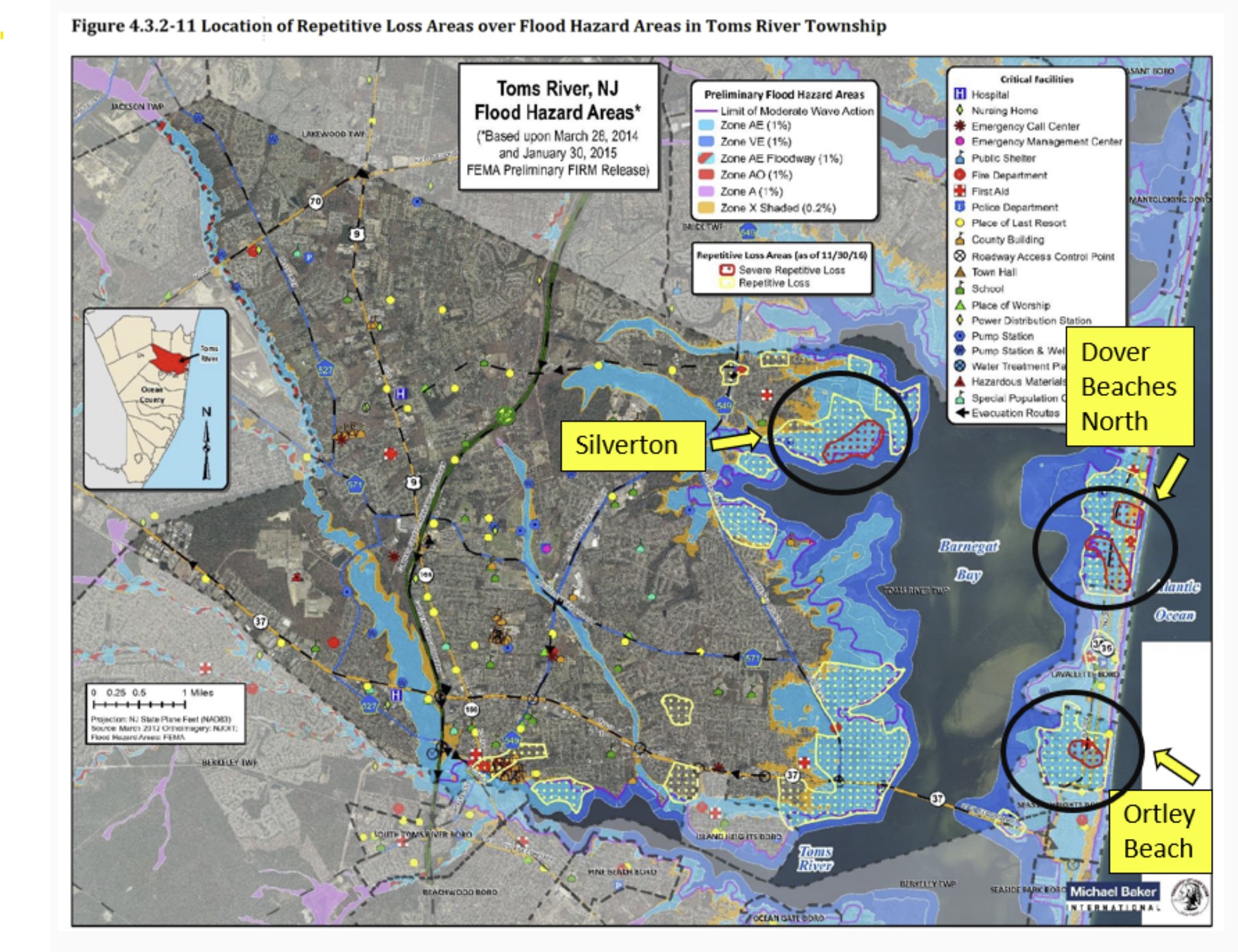


For more context, below we provide close-up maps of Ortley Beach neighborhood. The map was extracted from the 2014 Toms River Strategic Recovery Planning Report. The shaded purple area represents area affected by the Hurricane Sandy Storm Surge. Properties in yellow are those that were determined to be substantially damaged by FEMA.

About 4000 properties in Toms River were substantially damaged. Of these, 1,165 (or ~30%) were located in Ortley Beach.


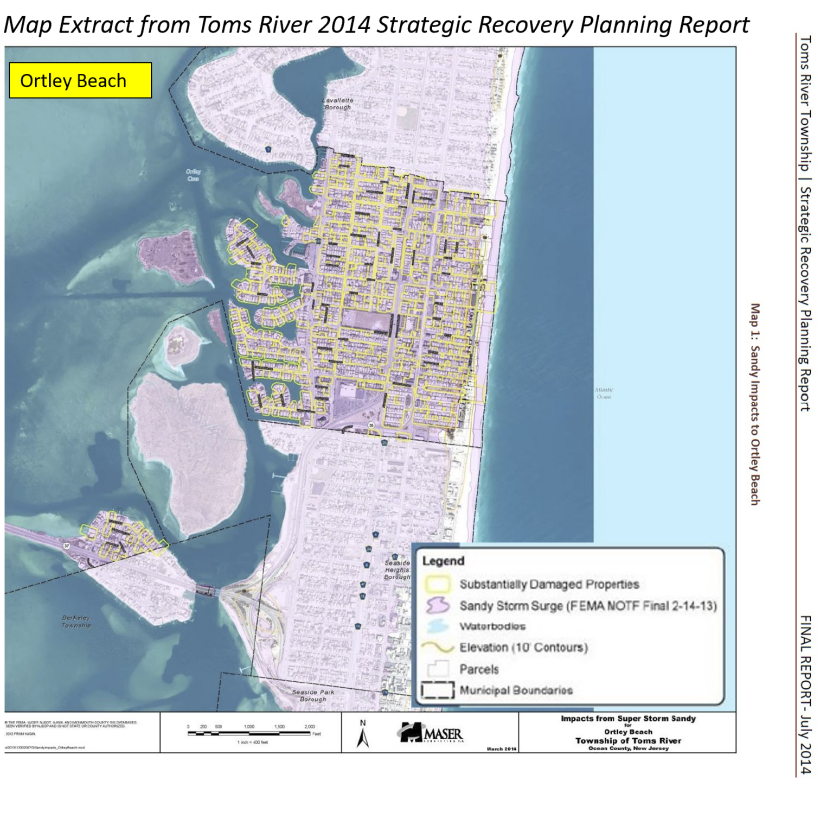


Below is a list of some strategies that have been identified to address the impacts of coastal flood risk, along with some basic diagrams roughly illustrating each strategy. Please indicate how strongly you agree or disagree with whether federal and state resources should be invested in each strategy within the context of Ortley Beach, NJ.

As you work through the survey, think through the pros and cons of this strategy as applied to your property or in your neighborhood. Given what you know about normal government budget constraints, what strategies would you prefer to see federal and state investments in?

*(Orient user with diagram and corresponding strategies in survey and discuss any questions).*

| Coastal Flood Risk Mitigation Strategy | Strongly disagree | Moderately disagree | Slightly disagree | Slightly agree | Moderately agree | Strongly Agree |
| --- | --- | --- | --- | --- | --- | --- |
| 1. No government interventions – let’s just seeing what happens and let the housing market respond to flood risk.   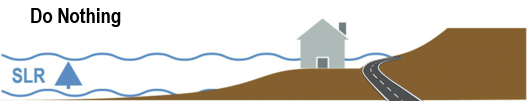 |  |  |  |  |  |  |
| 1. Build up land to make space for more housing.   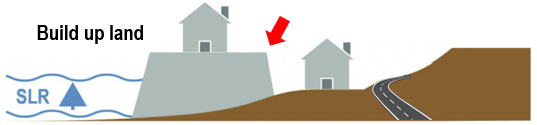 |  |  |  |  |  |  |
| 1. Build sea walls and raise the heights of existing sea walls to increase flood protection.   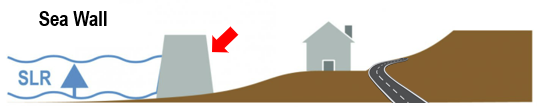 |  |  |  |  |  |  |
| 1. Purchase properties at market rate (buyout) and permanently relocate residents away from high-flood-risk areas. Restricting development in those areas.   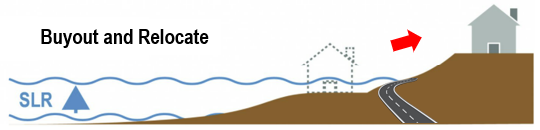 |  |  |  |  |  |  |
| 1. Elevate homes and buildings to decrease their risk of flooding.   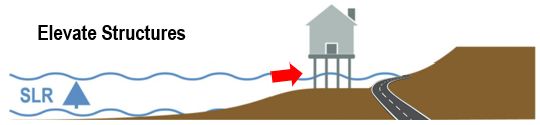 |  |  |  |  |  |  |
| 1. Replenish beaches with sand.   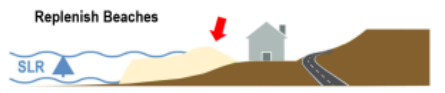 |  |  |  |  |  |  |
| 1. Restore natural systems like marshlands, and oyster beds to reduce flooding and wave impacts from storms.   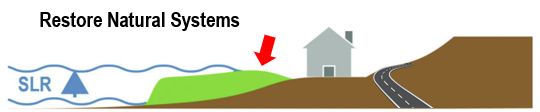 |  |  |  |  |  |  |
| 1. Elevate roadways   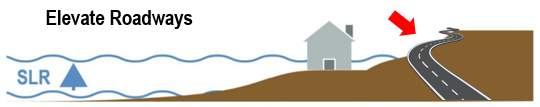 |  |  |  |  |  |  |
| 1. Increase insurance rates for homes in that are exposed or repeatedly flooded, to reflect flood risk more accurately.   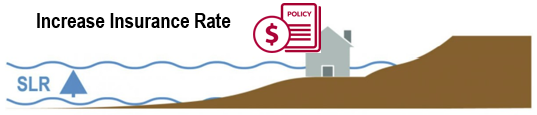 |  |  |  |  |  |  |
| **Other** (discuss)­­­ |  |  |  |  |  |  |

*(follow-up)* Now I’d like to ask you a few questions about your answers. Feel free to use the map to talk about the strategies in different contexts.

- 1. Looking at the strategies you agree with, do you have a preferred strategy or set of strategies for flood risk reduction in areas with repetitive loss? (*What do you like about these? Would they be more appropriate to certain locations?)*
  2. Now looking at the strategies you disagree with, *(Are there any/is)* that strategy(*s*) in that pile that are completely off the table for you for particular neighborhoods? *(Why? Why not?)*
  3. Given what you know about future flood risk, what strategies do you think are the most effective to mitigating risk in your neighborhood? *(Are there any thresholds beyond which certain strategies might not work? For example, do you think there might be tipping points where a strategy like elevating homes may no longer work? Do you think this strategy would be resilient in another storm like Sandy came along?)*
  4. Do you think any of these strategies would be especially controversial in your community? And why so?
  5. What strategies do you think would be best in the other neighborhoods with repetitive loss in Toms River –like Silverton, and Dover Beaches North?
  6. What additional information do you think decision-makers should have available to make an informed decision about the best strategy for your neighborhood?
  7. ***Time permitting, additional prompts on home elevations:***
     1. *In my research and observations driving around Toms River, I noticed that a popular property-level flood mitigation strategy is home elevations. Based on your knowledge, do most of the expenses for elevating homes come out of homeowner’s pocket? How much is subsidized?*
     2. *Are there any pros or cons to elevating homes as a flood mitigation strategy?*
     3. *Have you noticed changes to the character of communities where homes have been elevated?*

1. **Short Survey (*10 min*)**

To conclude, we ask that you please fill out this brief survey on worldviews, beliefs, and socio-demographics. The purpose of this brief survey is to gather information about your perspective or worldview on larger issues that are debated in society. We are interested in how people's worldviews, beliefs, and socio-demographics relate to their preferences for coastal flood management strategies.

For this section, your data will remain confidential following the research protocol for this study. All data will be aggregated and anonymized for publication. This survey should take about 5-10 minutes to complete. You may skip any questions if they make you uncomfortable. Completion of this survey is completely voluntary.

**4.1 Worldviews section**

We are interested in your perspective or worldview on larger issues that are debated in society. How strongly do you agree or disagree with each of these statements?

| Worldview statement | Strongly disagree | Moderately disagree | Slightly disagree | Slightly agree | Moderately agree | Strongly Agree |
| --- | --- | --- | --- | --- | --- | --- |
| 1) Sometimes government needs to make laws that keep people from hurting themselves. |  |  |  |  |  |  |
| 2) The government should do more to advance society's goals, even if that means limiting the freedom and choices of individuals. |  |  |  |  |  |  |
| 3) Our society would be better off if the distribution of wealth was more equal. |  |  |  |  |  |  |
| 4) We need to dramatically reduce inequalities between the rich and the poor, whites and people of color, and men and women. |  |  |  |  |  |  |
| 5) Discrimination against minorities is still a very serious problem in our society. |  |  |  |  |  |  |

**4.2 Beliefs section:**

We'd like to know what you think expert scientists believe about certain issues. For each statement below, please indicate whether you think that expert scientists agree, disagree, or are divided in their views about the statement:

| Statement | Most expert scientists agree. | Most expert scientists disagree. | Expert scientists are divided in their views. |
| --- | --- | --- | --- |
| 1) Sea levels are rising at an accelerating rate. |  |  |  |
| 2) Global temperatures are increasing. |  |  |  |
| 3) Human activity is causing global warming. |  |  |  |

**4.3 Socio-demographics section:**

4.3.1. How old are you?

- Under 18
- 18-24
- 25-34
- 35-49
- 50-64
- 65-74
- 45+

4.3.2. What is your gender?

- Female
- Male
- Other ______
- Prefer not to specify.

4.3.3. What is your dwelling status?

- Homeowner
- Renter

4.3.4. Which U.S. Census racial category best describes you?

- American Indian or Alaskan Native
- Asian / Pacific Islander
- Black or African American
- White / Caucasian
- Other

4.3.5. Are you of Hispanic, Latino, or Spanish origin?

- No
- Yes: Mexican, Mexican-American
- Yes: Puerto Rican
- Yes: Cuban
- Yes: Other

4.3.6. Is English your first language?

- Yes
- No

4.3.7. How many children under 18 live in your household?

- 0
- 1
- 2
- 3
- 4
- 5+

4.3.8. What is your highest level of education?

- Some high school
- Completed high school
- Some college
- Bachelor’s degree
- Some graduate school
- Master’s degree
- Doctoral degree

4.3.9. How many people earn income in your household?

- 0
- 1
- 2
- 3+

4.3.10. What is your annual household income?

- $0-$14,999
- $15,000 - $29,999
- $30,000 - $54,999
- $55,000 - $69,999
- $70,000 - $84,999
- $85,000 - $99,999
- $100,000 - $149,000
- $150,000 - $199,999
- $200,000+

1. **End (5 min)**

Finally, is there anything I didn’t ask you about or that you’d like to say regarding what we’ve discussed here today?

- (Prompt if time allows: For example, do you have any perspectives on how certain government programs are managed by FEMA, HUD, etc.?
- Do you think that federal dollars are being well-spent on flood risk management in the communities we discussed? Why or why not?
- In implementing projects, where are the hang up points for implementation? Which parts of the system: local, state, federal?
- Do you think that the projects are effective at mitigating future flood risk?
- Do you think that the way funds are distributed is fair?

Thank you very much for taking the time to talk with me. I am going to turn off the recording now.

# Interview Protocol: Decision-makers:

**NOTE:** This protocol was like the residents’ protocol, except that sections 1 and 2 are replaced by the following:

1. **Position and involvement in flood risk mitigation projects (Tailor as needed): *(10 min)***

Let’s get started with some general background questions on your position and your involvement in flood risk mitigation projects. I want to get a sense of the work that you do/ oversee.

- 1. How long have you been in your current position at (*mention agency and branch)*?
  2. What kinds of flood mitigation projects do you work on in (depending on position: National / State / Coastal New Jersey / Ocean County / Toms River)?
  3. If you could guess, how many flood-mitigation projects have you overseen in your current position?
  4. What repairs or modifications are typical for flooded or storm-damaged homes in the communit(y/ies) you work in?

# Supplementary tables and figures:

Table 1: Sociodemographic data for 21 local stakeholders that participated in the questionnaire:

| **Attribute** | **Frequency** |  | **Attribute** | **Frequency** |
| --- | --- | --- | --- | --- |
| ***Gender*** |  |  | ***Place of Residence*** |  |
| Male | 12 |  | Ortley Beach | 10 |
| Female | 9 |  | Toms River | 5 |
| ***Age*** |  |  | Seaside Heights | 4 |
| 25-34 | 1 |  | Lavalette | 1 |
| 35-49 | 2 |  | Long Branch | 1 |
| 50-64 | 8 |  | ***Education*** |  |
| 65-74 | 8 |  | Completed High School | 1 |
| 75+ | 2 |  | Some College | 2 |
| ***Race*** |  |  | Bachelor's Degree | 6 |
| White | 19 |  | Some Graduate School | 3 |
| Other | 2 |  | Master's Degree | 5 |
| ***English 1st Language*** |  |  | Doctoral Degree | 4 |
| Yes | 21 |  | ***Reported Income Range*** |  |
| No | 0 |  | Under $60,000 | 3 |
| ***Hispanic Origin*** |  |  | $60,000-$99,999 | 2 |
| Yes | 1 |  | $100,000-$149,999 | 3 |
| No | 20 |  | $150,000-$200,000 | 6 |
| ***Number of Children*** |  |  | Over $200,000 | 3 |
| 0 | 17 |  | NA | 4 |
| 1 | 3 |  | ***Tenure*** |  |
| 2 | 0 |  | Homeowner | 20 |
| 3 | 1 |  | Renter | 1 |

Panel 1: Select Socio-demographic characteristics for Ortley Beach and New Jersey (2010-2022):


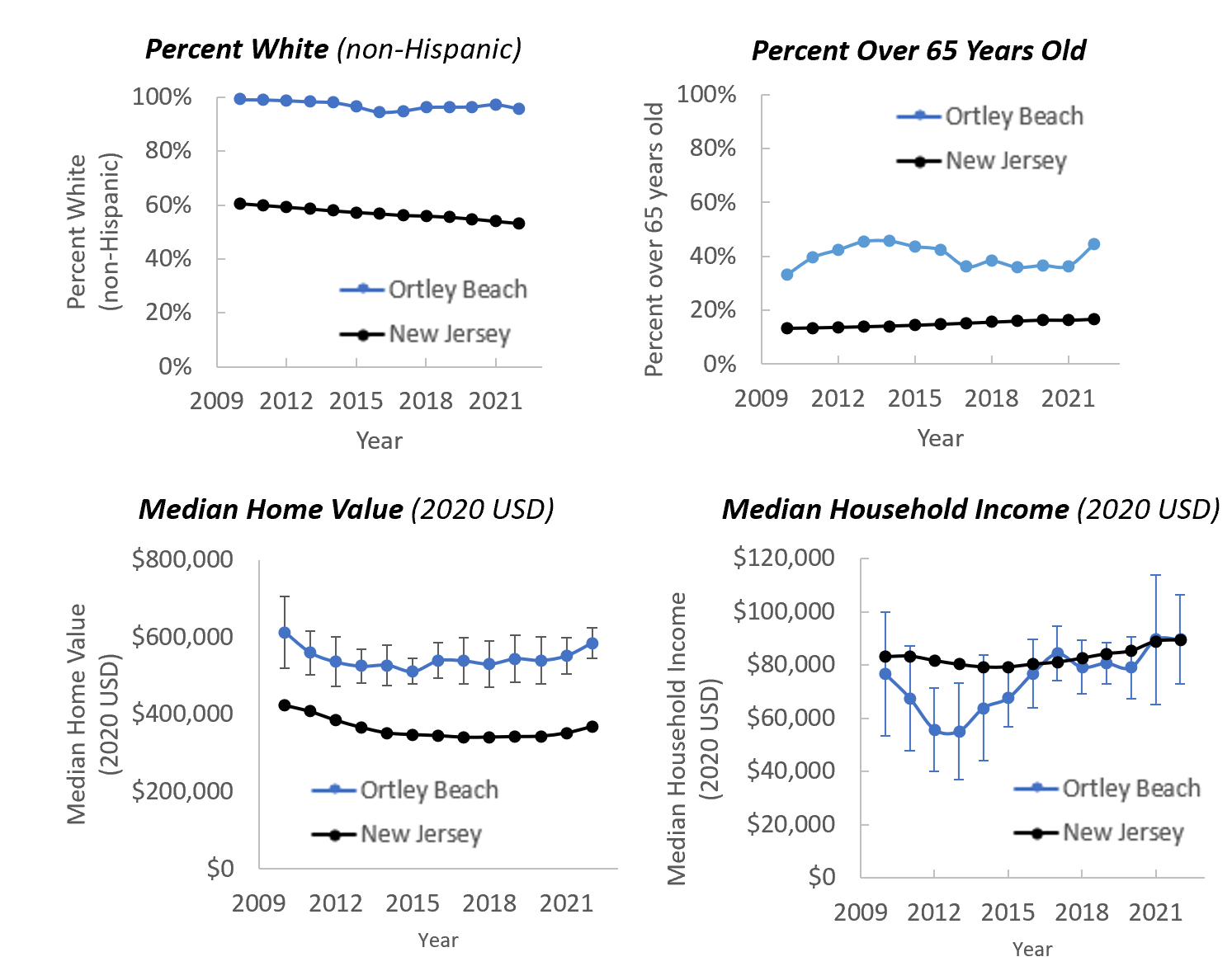


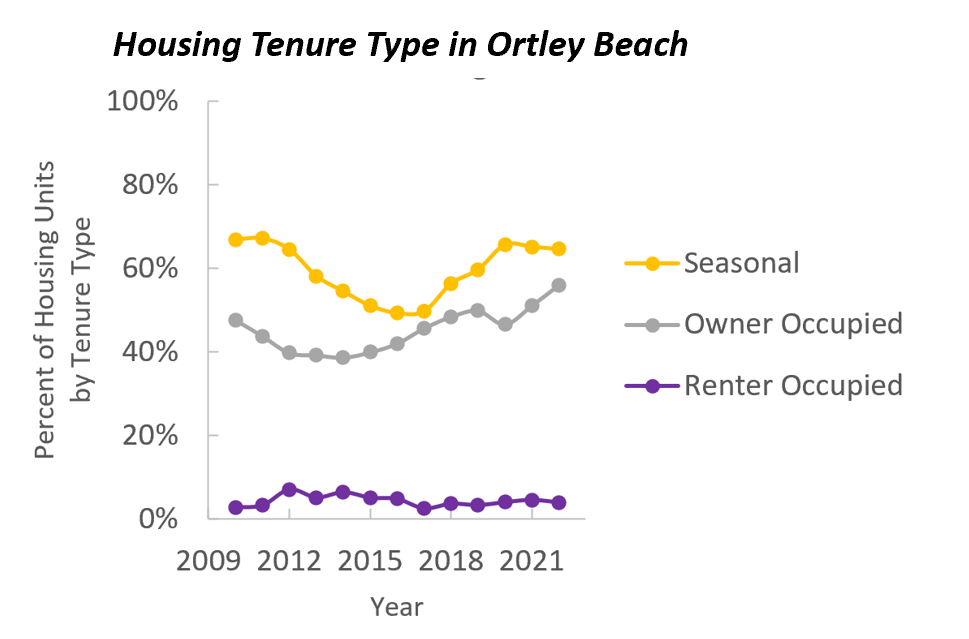


Table 2: Values Articulated by Respondents in Relation to CRRS:


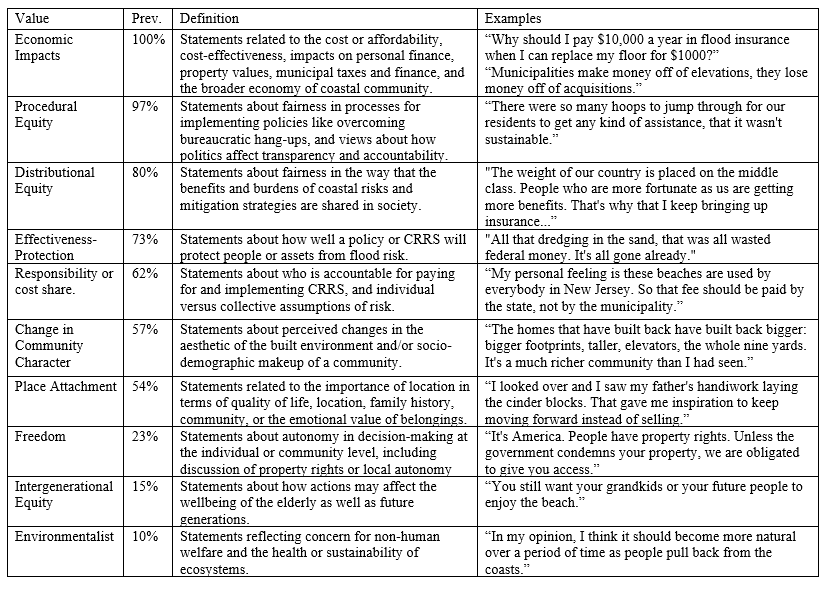


Figure 1: Map of Ortley Beach, Toms River, New Jersey
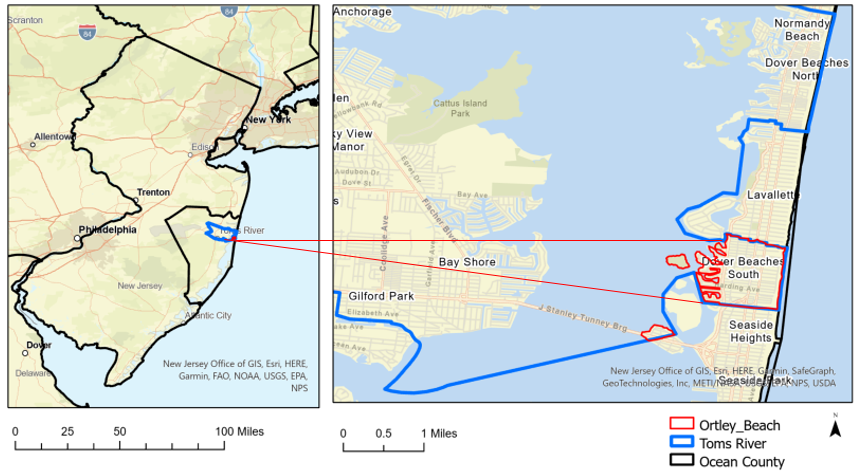

Supplement: Supplementary file 1 — Supplementary Materials: risa70091‐sup‐0001‐SuppMat.docx [file RISA-45-3519-s001.docx]
